# Synthesis and application of pillared clay heterogeneous catalysts for wastewater treatment: a review

**DOI:** 10.1039/c7ra12924f

**Published:** 2018-01-30

**Authors:** Jeffrey Baloyi, Thabang Ntho, John Moma

**Affiliations:** Molecular Science Institute, School of Chemistry, University of the Witwatersrand P/Bag 3, WITS 2050 Johannesburg South Africa john.moma@wits.ac.za; Advanced Materials Division Mintek, Private Bag X3015, Randburg 2125 South Africa

## Abstract

The use of pillared interlayered clays (PILCs) as heterogenous catalysts in wastewater treatment technologies, particularly advanced oxidation processes (AOPs), is gaining popularity for the treatment of refractory wastewater effluents. The recent literature involving these solid materials is reviewed, with more focus on studies that aim at reducing the synthesis costs and escalating the synthesis process to industrial scale. Their role as active solid materials in the AOPs such as photocatalysis, catalytic wet peroxide oxidation (CWPO), the Fenton process and catalytic wet air oxidation (CWAO) of refractory organic compounds in polluted aqueous streams is also reviewed. These processes are evaluated to evidence their main direction for future research, particularly with reference to possible industrial use of these technologies to treat refractory organic wastewater using pillared clay-based catalysts. The pillared clay catalysts demonstrate good application prospects for the removal of refractory wastewater effluents using AOP technology. The reviewed studies suggest that the photocatalytic process is useful in low concentrations of these compounds, while CWPO, the Fenton process and CWAO are recommended for higher concentrations. However, catalyst development to reduce the severity of oxidation reaction conditions, with focus on the low cost, catalyst stability, reusability and environmental friendliness are the key aspects to be addressed by future research work.

## Abstract 2

The use of pillared interlayered clays (PILCs) as heterogenous catalysts in wastewater treatment technologies, particularly advanced oxidation processes (AOPs), is gaining popularity for the treatment of refractory wastewater effluents.

## Graphical abstract

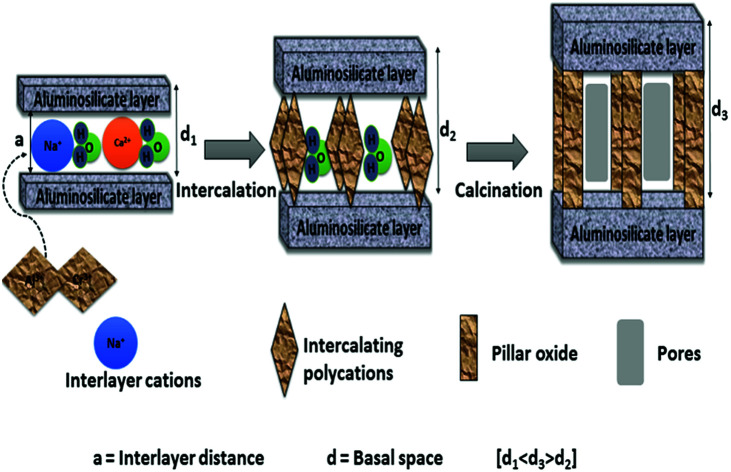

## 1. Introduction

The use of green chemistry and green engineering intimates the design of chemical products, processes that reduce or eliminate the use or generation of hazardous substances from all sources and overall cost minimization.^1^ Moreover, the minimization of toxicity and hazards as well as the maximization of safety practices are important considerations. When these terms are applied to the performances of individual chemical reactions, chemical processes, and chemical synthesis plants, they deal with the same issues. Advances in both green chemistry and green engineering address the presence of toxic substances in the environment, hazards associated with global issues such as climate change, energy demands, global warming, availability of a safe and adequate water supply and interest in sustainability. These concepts have then become very popular. In recent years there has been an increasing interest in the use of pillared clays minerals, also called pillared interlayered clays (PILCs), as green heterogeneous catalysts in the wastewater treatment.^2–4^

Applications of clay minerals have attracted a lot of interest due to their potential use in heterogeneous catalysis.^5,6^ However, clay minerals inherently have the shortcoming of poor access to their active sites which decreases their performance for catalytic reactions. Nevertheless, modification of natural clay minerals by pillaring using suitable pillaring agents has emerged as a promising technique because it allows for opening of the clay layers, producing high resistance and thermal stability, increased porosity, surface area and basal spacing as well as other physicochemical characteristics.^7,8^ The pillaring method is based on a mechanism of cationic exchange in which robust inorganic molecules are introduced in the interlayer space of the clay minerals forming oxides strongly bound to layers of the minerals.^9^ The hydroxyl polycations of polynuclear metals, such as Al, Fe, Zr, Cr and Ti, are types of inorganic pillaring agents that have been widely studied. Numerous studies have discussed these solid materials in details and are cited in the literature.^10,11^ Incorporation of a second pillaring agent (*e.g.*, M = Zr, Fe, Cr and Ti) in the Al inorganic agents to create mixed oxide PILCs have emerged.^11,12^ The general preparation process of mixed oxides pillaring agents (*e.g.* Al/M) is similar to the preparation of a single metal oxide agent, except that the base solution (*e.g.* NaOH or Na_2_CO_3_) is titrated into the Al(NO_3_)_3_ and M pillaring agent solutions with different Al/M molar ratios. Incorporation of a second metal oxide is reported to further improve the structure by increasing the pore volume and specific surface area (200–300 m^2^ g^−1^), thermal stability and acidity of the catalyst compared to single metal oxide PILCs.

The conventional preparation of PILCs consists of two important stages: the preparation of the metal oxide pillaring agent in solution and the mixing of pillaring agent with a suspension (typical 2% in weight) of clay in water.^13^ This method involves long periods of time and large amount of water, and such requirements represent a considerable shortcoming when trying to scale-up the pillaring method to industrial scale.^14^ Therefore, the use of PILCs as heterogeneous solids catalysts has not been escalated to commercial levels, due to the fact that it has been challenging to perform the conventional laboratory synthesis method at industrial scale.^15^ In order to reduce the large volume of water required, currently the pillaring method is focused on application of concentrated clay suspension, dry clay and concentrated pillaring agent solution.^4^ Similarly, direct addition of clay to pillaring solutions decreases the time required for the preparation process and methods such as ultrasound,^4^ microwave^16^ and one-step high-temperature synthesis^10^ have recently emerged as a powerful and green approaches for synthesis of PILCs. All these methods, obtain solid materials with comparable characteristics to those of the solid materials synthesized by means of the conventional method, however, within shorter time for the process. Moreover, several studies reported that when using the conventional method, the aging time may vary by hours or days (1–5 days) depending on the pillars.^17–19^ With ultrasound, microwave and one-step high-temperature synthesis methods, the aging and ion exchange process occur in minutes (5–30 minutes), therefore facilitating its extension to an industrial scale. However, commercialization of PILCs catalysts is not only influenced by the optimization of preparation parameters but also by the ability to shape the powder material into conformed products such as pellets, monoliths and agglomerates, *etc.* The prepared catalytic solids materials are expected to keep their chemical properties and catalytic activity as well as maintain their stability during the reaction process. Mohino *et al.*^20^ and Sanabria *et al.*^21^ used the extrusion method to manufacture raschig rings, pellets and monoliths that involve pillared bentonite clay minerals. Numerous studies available in literature on the manufacture of extrudates are related to ceramic monoliths such as sepiolite, cordierite, mullite, zirconia, titania, α- and γ-alumina and mixture of these materials.^22,23^ However, few studies have been reported relating to fabrication of extrudates based on pillared clay catalysts, which remains an important aspect for PILCs to be used at an industrial scale level.^21^ Therefore, there is a great need for creative strategies which should enable PILCs to be engineered to “designer catalysts” for applications in green, eco-friendly and sustainable wastewater treatment processes. Wastewater contaminated by phenol and its derivatives is one of the most common and essential representatives of refractory organic wastewater produced by industrial methods such as petrochemical, paper, paint, fabric and chemical industries, because of its high toxicity even at low concentrations. Phenol is listed as a primary pollutant by WHO (World Health Organisation). It is considered toxic at concentration above 2 mg L^−1^, and when it is present in natural water streams it can lead to the formation of harmful chlorine solutions during disinfection by chlorination.^24^ Typical concentrations of phenolic compounds in these industrial wastewaters are in the range of 200–1500 mg L^−1^ whilst the Environmental Protection Authority's limit for wastewater disposal discharge is 0.5 mg L^−1^ for surface water and 1 mg L^−1^ for sewerage water.^25^ In the past years, wastewater containing phenol has received increased attention because of its extreme toxicity to aquatic life and resistance to biodegradation.^24,26^ Consequently, with regards to human health and the environment, it is crucial to purify water contaminated with phenol and its derivatives, which can be present in almost all industrial wastewater streams.

Conventional processes for removal of phenol from industrial wastewaters include biological, adsorption, extraction, chemical oxidation, electrochemical methods and irradiation *etc.* The above mentioned methods suffer from serious shortcomings such as high costs, incompleteness of purification, formation of hazardous by-products, low efficiency and applicability to a limited concentration range (these methods are not suitable for treating moderate to high concentration organic pollutants such as phenol).^27,28^ Therefore, it is of great importance to find innovative and simple methods and approaches to tackle environmental problems associated with wastewater contaminated by phenol. The use of advanced oxidation processes (AOPs) in the presence of PILCs to treat environmental contamination by refractory organic wastewater containing phenol is one of the most promising methods that have received increased attention in recent years.^4,21,29^ AOPs, especially using PILCs catalysts as cheap natural materials are able to remove organic contaminants from aqueous effluents without high costs and complex technical requirements. Catalytic wet air oxidation (CWAO), catalytic wet peroxide oxidation (CWPO), Fenton-like process and photocatalytic treatment of wastewater are some of the successful AOPs processes, utilising catalysis for the removal of pollutants in industrial wastewater in the presence of an active catalyst.^30^ AOPs are all based on the high reactivity together with low selectivity of attack displayed by hydroxyl radicals (HO˙). AOPs may achieve full mineralization of most of the refractory organic contaminants to CO_2_ and H_2_O, or at least partial degradation of the substrates releasing less harmful by-products.^28^ Therefore, these processes play crucial roles as green engineering processes in the wastewater treatment of moderate to high concentration of organic contaminants such as refractory organic pollutants.

In this context, this article aims at undertaking a systematic review of heterogeneous pillared clay catalysts syntheses processes based on recent and previous studies. The green route syntheses, modification and applications of the pillared clays catalysts in AOPs have been discussed in detail. The synthesis and application of pillared clay catalysts for refractory organic wastewater treatment will be reviewed with focus on aspects that have not yet been comprehensively reviewed such as a new insight into understanding the role of new robust sustainable green synthesis methods and their eco-friendly approach towards commercialization.

## 2. Synthesis of pillared interlayered clays (PILCs)

Pillared inter-layered clays (PILCs) are an imperative group of microporous inorganic solids with great catalytic potential for applications in catalytic processes.^31^ PILCs have attracted increasing attention, particularly from industry, due to their microporous nature and wide range of applications such as catalytic oxidation, hydrogenation, dehydrogenation, hydroxylation, esterification, catalytic cracking, and others.^13^ All of the above-mentioned reactions are potentially interesting in general green engineering industrial methods and green chemistry techniques.

The phenomenon used in the synthesis of pillared clays is the ion exchange of interlamellar cations by bulky cationic species that act as supports to keep the structure open. It is well known that only swelling clay minerals that are capable of cation exchange such as smectite group (bentonite) can be pillared. The first step in the pillaring of PILCs involves the synthesis of the pillaring agent. In this step the pillaring solution undergoes hydrolysis, polymerization and complexation with the anion in the solution.^32^ The hydrolysis conditions such as temperature, pH and aging time, play an important role in the formation of PILCs. In the case of Al^3+^ polyoxocation, a base (NaOH or Na_2_CO_3_) is added to AlCl_3_ or Al(NO_3_)_3_ solutions with OH/Al^3+^ ratios up to 2.5. Kloprogge^17^ analyzed the polyoxocation complex produced during the process and is thought to be the Keggin ion [AlO_4_Al_12_(OH)_24_(H_2_O)_12_]^7+^. The conventional process involves prolonged times and this makes the pillaring process not applicable in an industrial scale. This stage of the synthesis process has gained considerable attention with many studies aimed to reduce time and energy costs by finding ways to economize the process for commercial feasibility. Martinez-Ortiz *et al.*,^33^ Olaya *et al.*^12^ and Sivaiah *et al.*^34^ used microwave radiation during the synthesis of pillaring solution with a reduction in the synthesis time needed from several hours to less than 30 minutes. Tomul^4^ recently investigated the use of ultrasound treatment during the preparation of iron–chromium pillaring solution. The Fe/Cr pillaring solutions was aged for less than 20 minutes compared to hours or days required when conventional method is used. The characteristics and catalytic behavior of the resulting pillared solids confirmed the beneficial effect of using ultra sound treatment.

The synthesis of the pillaring agent is followed by slow addition of the polyoxocation pillaring solution to the clay suspension. This allows the interlayer inorganic cations in the clay to exchange with the polyoxocation in solution through intercalation.^6^ This results in up to five times increase in basal spacing (*d*_001_) of the clay mineral, which is generally determined by XRD. When the conventional method is used, this process can take up to hours or days to complete depending on the pillars, as the pillaring solution is added drop-wise and the mixture is stirred for at least hours with heat supplied.^10,15,35–37^ Although this method has been successful in laboratory synthesis of pillared clays, it is not an ideal method for industrial scale production because of the large amounts of water and heat required. However, using an ultrasound or a microwave treatment method, the ion exchange occurs in minutes. Duong *et al.*^38^ studied the intercalation of montmorillonite and found ultrasonic treatment to reduce the intercalation time from several hours with the conventional method to less than 20 minutes. They however found prolonged ultrasonic treatment to result in partial destruction of the pillared structure. The obtained solids were comparable in physical and catalytic properties to that from the conventional method. Most importantly, no heat was needed in the pillaring process, with the added advantages of cost saving and minimal safety risks. Lastly, the amount of water used and space were minimized by use of a more concentrated clay suspension compared to conventional method. Therefore, the synthesis of PILCs on a commercial scale can be attained since the intercalation of the pillaring complexes can be achieved within a short period of time without losing catalytic activity. Numerous studies also concluded that ultrasound treatment method can enhance and promote dispersion of the active metal on the carrier, therefore, improving the catalytic performance of the pillared clay catalysts.^12,19,39,40^

The application of microwave radiation has also been reported to reduce the time of the synthesis during intercalation stage. Olaya *et al.*^12^ reported the synthesis of Al pillared clay catalyst using a concentrated suspension of the pillaring solution and the clay with microwave radiation. It was found that the solids synthesized using microwave irradiation exhibited better characteristics than those of solids synthesized by the conventional method. They concluded that the synthesis of Al pillared clays using concentrated suspensions of clay and microwave provided advantages at the industrial level compared with the conventional method. This can be attributed to the fact that it decreases the time and volume of water required in the process, and also generates solids with high catalytic activity. A number of studies also reported PILCs materials synthesized using microwave irradiation with characteristics that can be matched with those of PILCs materials synthesized by means of the conventional methods.^15,36,41,42^

Another problem which hinders the commercialization of PILCs at industrial scale is that the clay dispersion is diluted by the use of high volumes of water during the synthesis process, and addition of pillaring solutions to the clay suspension takes a long time. As reported by numerous studies water consumption can be significantly reduced by using concentrated clay suspensions and dry clay.^14,37,43^ It has been reported that starting with dry clay generated the highest degree of modification. This could be attributed to strong interaction of vibrating molecules due to microwave radiation and generation of higher resistance in the medium, which also results in higher dielectric heating than in a diluted system.

**Fig. 1:**
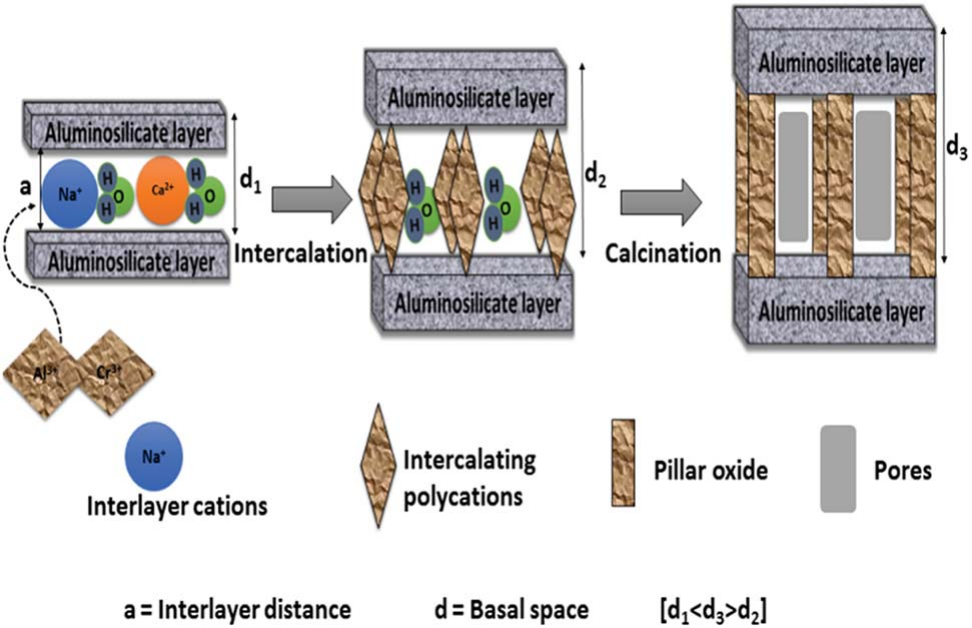
Schematic representation of clay pillaring process (basal spacing; *d*_1_ < *d*_3_ > *d*_2_).

The final step is the washing and then calcination of the dried obtained solids. This step results in the dehydration and dihydroxylation of the polyoxocation where it changes to stable oxide clusters. This step is also referred to as thermal activation. Thermally stable porous solids are obtained after calcination. During calcination the bond between the interlayer species and clay layers is thought to shift from ionic to near covalent which results in the stabilisation of the porous network, by converting hydroxide pillars to stable oxides.^6^ The basal spacing may decrease, but should not collapse due to calcination at high temperatures. It is also suggested that the intercalation species must not fill the entire interlayer clay space in order to have accessible porosity with high specific surface areas.^44^ Excess ions such as chlorides or nitrates that can still be found in the solid materials prior to calcination can also be removed in this step. Due to the re-suspension of the separated clay minerals in high purity water, the washing step is also considered as more time consuming step. The washing step is repeated several times until the chlorides or nitrates ions are completely removed from solid clay materials. The high volume of water required during the washing step hinders the industrial scale-up of the solid clay materials. The effect of washing step on Al-PILCs was investigated by Thomas and Occelli.^45^ The samples which were repeatedly washed up to 4 times with 400 cm^3^ of high purity water and the samples with no washing were compared. The XRD results revealed a broad weak peak for the unwashed sample, which then shifted to 18.9 Å with the first washing, and then became sharper with consecutive washing. Their study concluded that the initial cations present in the interlayer space were formed “*in situ*” by base hydrolysis of the different oligomers present instead of Keggin. Therefore, the washing step is found to be necessary to form stable Al-PILCs as their stability is linked to the creation of Keggin ions. Molina^46^ was the first to publish a method in which concentrated (40% w/w) clay slurries, contained in dialysis bags, were equilibrated in an Al-pillaring solution. Subsequently, del Riego *et al.*^47^ used the same method to pillar concentrated (10% w/w) raw and purified bentonite clay and found that the use of dialysis membranes aided the recovery of the concentrated suspensions of pillared clay materials and the number of washing cycles was decreased. However, the procedure was time-consuming and high volumes of solutions were required since the pillaring solution remained dilute. The use of dialysis compared to conventional washing methods was examined by Aceman *et al.*^48^ who found that at first Al was adsorbed into the interlayer space either in a monomeric state or as small oligomers. The hydrolytic oligomerization to form Keggin ions with these species were achieved after a number of days, given that the excess ions such as Al, Cl and Na were removed through washing or dialysis. Intercalated clay sample inside the Visking dialysis bag was put in double distilled water for 7 days at room temperature in the dialysis experiments. The thermal stability of dialyzed intercalated montmorillonite clay samples was similar to non-dialysed samples, while intercalated laponite and hectorite clay samples resulted in poor thermal stability for both dialyzed samples and the washed samples. Both dialyzed intercalated beidellite and saponite clay samples demonstrated high thermal stability as revealed by more intense sharper XRD peaks compared to those washed 4 times. Sampieri *et al.*^49^ investigated the effect of washing. For solids samples washed with a large volume of water (2 L g^−1^ of montmorillonite clay), the larger silica species were expelled irrespective of the synthesis method used in the study. The study concluded that the washing step is critical in synthesis methods, as it determines the interlayer space, thermal stability and the porosity of the pillared montmorillonite. However, the pillared structures were stable up to 600 °C, but collapsed at 700 °C. Fig. 1 displays schematically the pillaring process.

In general the current problems associated with the above preparation procedure are long synthesis periods and high energy costs, high volumes of water usage and mixing of clay solutions.

Therefore, due to the above-mentioned problems PILCs have not been applied as commercial catalysts. This is because of the difficulties in escalating the laboratory scale developed pillaring process to an industrial scale. In order to produce PILCs at an industrial scale, the above mentioned procedure must be simplified.

## 3. Recent advances in the synthesis of PILCs

Commercially, when huge amounts of PILCs are required to be synthesized, conventional methods become uneconomical, since enormous equipment as well as the use of large volumes of water are required in the process.^37^ The synthesis of pillared clays requires several optimizations to enable them to be applied at industrial scale. These include the use of raw natural clays without further purification^4,12,13^ and the use of advanced green chemistry synthesis methods such as ultrasound, microwave radiation and one-step high-temperature high-pressure syntheses.^10,14,15,50^ In addition, in order to improve the structure and acidity of mononuclear inorganic PILCs, the addition of a second pillaring agent such as Cr, Zr, Fe and Ti to the Al pillaring agent solution is also recommended.

The effect of addition of second metal oxides (*e.g.* M = Fe, Zr, Cr and Ti, *etc.*) to the Al inorganic pillaring solution has been extensively investigated. Carriazo *et al.*,^44^ Molina *et al.*^51^ and Zuo *et al.*^52^ obtained solids with higher surface areas (increase from 200 to 300 m^2^ g^−1^) and pore volumes than single metal oxide (Al-PILCs). However, the drawbacks of the synthesis methods of the pillaring agent solutions are not considerably improved.

The removal of traces of organic matter, iron oxides and soluble salts from the natural clay before pillaring has also been investigated. Prior to pillaring process high content of iron oxides in the surface are generally removed from raw natural clay due to reports that they affect the synthesis process, textural properties and catalytic activity of PILCs as a result of the fact that excessive iron oxides content may change the colloidal and rheological behavior of both the suspended natural clay and the cation exchange capacity of the mineral clay present.^53^ For more cutting-edge green chemistry processes some excellent properties of shaped PILCs such as extruded solids, pellets, agglomerates and monolith macro-structures are preferred. However, excessive iron oxides content may reduce both the thermal and mechanical stability of the solid materials. The PILCs catalysts' acidic and redox properties could be modified due to the excessive iron oxides content present in the raw natural clay. However, the use of purified clays is not preferred for the industrial scale process due to the fact that this cation exchange involves the use of high volumes of water and sodium salts. Therefore, the use of raw natural smectite clays with high swellability such as sodium bentonite is preferred, as it makes it easier to incorporate large metal polyoxo complexes.

The methodology for the synthesis of PILCs with single and mixed metal oxides that was developed by the use of ultrasound, microwave and one-step high-temperature high-pressure method during aging of the pillaring solution and interaction with the powdered raw natural clay has been reported to decrease the synthesis time as well as the volume of water used in the pillaring process by approximately by 90 and 95%, respectively. At an industrial scale, this leads to a significant reduction in the cost of the synthesis processes.^10,12,13,15^

Several studies^4,13,15,35,40,54,55^ found the use of ultrasonic treatment to reduce the synthesis time of PILCs to less than 20 minutes. The obtained PILCs had a basal spacing ranging from 18 to 20.6 Å and a BET surface area in the range of 134 to 362 m^2^ g^−1^. In order to test their thermal and hydrothermal stabilities, the PILCs were heated at temperatures ranging between 400 to 900 °C in a muffle furnace for 2 to 8 hours. The PILCs intercalated by the conventional method were exposed to comparable conditions. The results showed that the structure of PILCs prepared by conventional method collapsed at around 600 to 700 °C while the PILCs prepared by ultrasonic treatment partially maintained their structure at 900 °C. This stability was attributed to the uniform pillaring obtained by the use of ultrasonic treatment. Ultrasonic treatment did not change properties such as acidity and catalytic activity of the PILCs but was found to improve the incorporation of pillars of larger size and higher homogeneity into the clay, compared to the conventional synthesis. The ultrasonic treatment method offers several advantages to the scale up of these PILCs. Firstly, it reduces the time required from several hours or days to less than 20 minutes. It also requires no heat for the process, thereby offering a considerable saving in energy costs. In conclusion, the dry raw natural clay can be directly added to the pillaring solution and the resulting clay suspension can be highly concentrated compared to conventional methods, therefore using less water, shorter period and volumes.

The use of microwave irradiation has been reported by various authors to also speed up the PILCs synthesis process^12,16,18,33,56,57^ requiring less than 15 minutes for the intercalation. Fetter *et al.*^56^ investigated ways to accelerate the intercalation step during the synthesis of PILCs by using microwave irradiation. In their study 10 wt% clay suspension was slowly added to Al chlorohydrate to give an Al/clay ratio of 5 mmol g^−1^. The solid sample was sealed and exposed to microwave irradiation for different time periods and then pillared by the conventional method. It was found that the specific surface areas of the solids prepared using microwave irradiation increased by 20–30% compared to samples synthesized by the conventional method. The microwave irradiation time was found to have little effect on the specific surface area, attaining 340 m^2^ g^−1^ in 5 minutes of irradiation compared to 18 hours using the conventional method. Similarly, Fetter *et al.*^57^ synthesized PILCs solids materials *via* microwave irradiation for 7 minutes using highly concentrated starting clay slurry of 50 wt% and obtained a specific surface area of 331 m^2^ g^−1^, which was 27% higher compared to solids samples synthesized by the conventional method. Olaya *et al.*^12^ developed a novel methodology for the synthesis of composite Al/Fe and Al/Fe/Ce pillared clays. The starting raw clay was added directly to pillaring solution and microwave radiation was applied during both the synthesis of the pillaring solution and in the intercalation process. The amount of water used and the processing time were reduced by 90–95%, offering new routes for the industrial scale up of the synthesis of the PILCs. The most promising materials prepared by the methodology proposed in their study, were synthesized using between 50 and 90% less Fe and Ce than the PILCs synthesized by the conventional method. The XRD patterns showed more intense and homogeneous (001) diffraction lines with a high content of both Al and Ce while BET results showed higher specific surface areas compared to PILCs prepared *via* conventional methods. Their activities and selectivities in the oxidation of phenol in wastewater were found to be superior compared to PILCs from conventional preparation method with higher Fe content. A lower Ce was found to have a positive effect on the incorporation of Al, the homogeneity of the (001) diffraction lines, the high specific surface area and high catalytic activity, demonstrating that the catalytic activity of the solid materials depends on the success of the pillaring process. The study by Sanabria *et al.*^16^ also found that the synthesis of PILCs composites Al/Fe and Al/Ce/Fe by microwave radiation in a concentrated medium resulted in a 90–95% reduction in amount of water used as well as the intercalation time by 70–93%. The PILCs synthesized by this method were comparable in their catalytic activity and selectivity to those from the conventional synthesis procedure in a dilute medium. The active phase was reported to be more stable during the reaction and the leaching of Fe was less than 0.18 mg L^−1^.

Recently, Ding *et al.*^10^ proposed a method for the synthesis of AlCr pillaring agents *via* one-step high-temperature high-pressure hydrothermal procedure, and the composite of Al/Cr-PILCs were synthesised using ion exchange. Variations in synthesis parameters such as reaction temperature, Al/Cr molar ratio and reaction time were found to affect the structure and textural properties of Al/Cr-PILCs. The specific surface area, pore volume, and maximum basal spacing of Al/Cr-PILCs reached 266 to 362 m^2^ g^−1^, 0.16–0.22 cm^3^ g^−1^, and 2.06 nm, respectively, after 4 hours of calcination at 550 °C. The as-synthesized composite Al/Cr-PILCs were porous materials with outstanding structures and performance compared to mononuclear Al-PILCs and Cr-PILCs. The synthesis procedure significantly reduced the number of steps as well as the amount of materials used compared to conventional methods. Therefore, this new proposed one-step method can be extensively applied to the synthesis of PILCs.

## 4. Synthesis of PILCs extrudates and monoliths

**Fig. 2:**
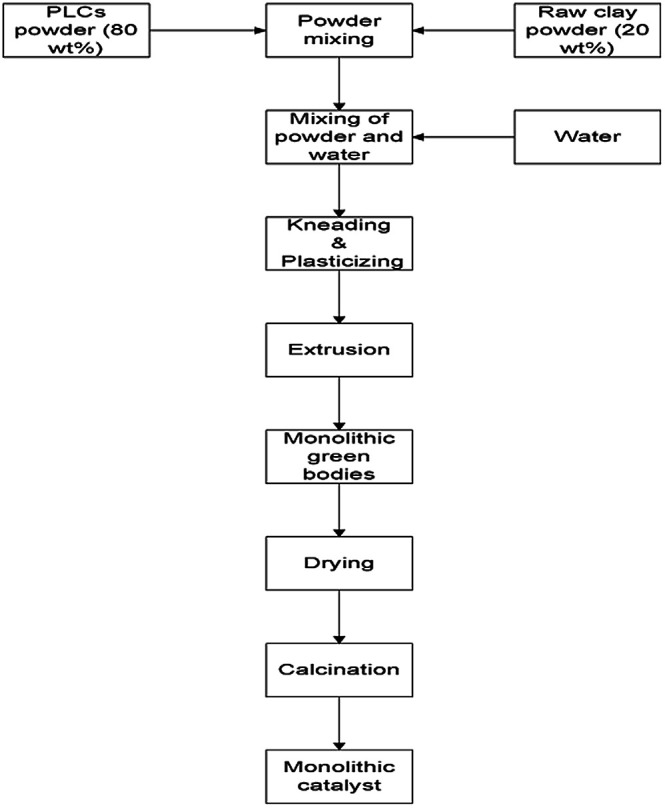
Schematic representation of extrusion process.

The scale up of catalysts based on PILCs to a commercial level for industrial applications also depends on the ability to match the synthesized powder pillared clay materials from laboratory scale to commercial shapes such as pellets, Raschig rings, monoliths *etc.* These materials after formation should keep their chemical properties, catalytic activity and stability during the reaction similar to the powder pillared clay materials. The synthesis of pillared bentonite clay materials with different shapes such as pellets, Raschig rings and monoliths has been achieved *via* extrusion techniques.^20,21,58^ There are four steps that are involved in the extrusion process; the mixing and kneading of powder components in dry state is considered as the first step. In this step powder materials are selected based on the final solid material's chemical composition. In order to impart plasticity and lubrication to the solid mixture throughout extrusion, liquids such as water and plasticizers are added in the second step. In the third step, high-shear mixing, extrusion and thermal processing are applied to fix the solid structures and impart to the green body mechanical strength. The synthesis schematic representation of the extrusion process is shown in Fig. 2.

To date, most of the studies on the manufacture of monoliths, investigated the catalysts materials based on conventional supports such as alumina,^59^ cordierite,^60^ activated carbon,^61^ zeolite,^62^ titania^63^ and mixtures of these materials.^23^ On the other hand, in developing new catalyst systems, it would be appropriate to explore the opportunity of synthesizing monoliths materials with stable, less expensive and high surface area materials. Generally, monolithic catalyst structures have several advantages over conventional solid particle catalysts. These include low pressure drop, high mass transfer, short internal diffusion distance, very low bed flooding, high liquid velocity, good thermal and mechanical properties, simplicity in scale up, *etc.*^23^

Sanabria *et al.*^21^ developed extrudates on Al/Fe and Al/Ce/Fe pillared bentonite composites which were shaped as pellets, Raschig rings and monoliths and the precursor materials were Na- or Ca-bentonite clay as binder. The catalytic activities of these composite materials were investigated for the oxidation of phenol in wastewater. The mechanical resistance of the extrudate materials depended on the calcination temperature and at 500 °C, good mechanical strength and sufficient stability to immersion in water were achieved. The as-synthesized extrudate materials retained the structural and textural properties of the powder materials. However, there was a decrease in specific surface area and pore volumes, which was reported to be due to the added bentonite as binder. The formed extrude materials preserved the catalytic properties of powder form. However, longer reaction times were required to accomplish a comparable phenol oxidation activity as well as TOC reduction, likely due to the diffusional limitations inherently present in the extrudates.

Mohino *et al.*^20^ investigated features linked to the composition of the paste in the synthesis of monoliths of Al-PILCs as well as the effect of parameters such as temperature and pH on the physicochemical properties of the synthesized monoliths. In their study PILCs were introduced as starting material to prepare monoliths, and described the manufacturing conditions under which the resulting extruded materials offer the suitable combination of both physicochemical and mechanical properties as to be used as catalyst materials or catalytic supports. This study showed that extruded monolithic honeycombs with high surface area can be easily synthesized using Al-PILCs as starting raw material. The original properties of the PILCs were improved by the obtained monolith catalysts. These improved properties such as high surface area, acidity and reactivity, a good mechanical strength and resistance to pH and temperature makes the PILCs to be able to be applied as green catalyst in catalytic processes. It was therefore concluded that the Al-PILCs can be regarded as a competitive material for any other high specific surface area materials for instance zeolites, to conform monoliths either as catalysts or as active phase carriers in a number of catalytic processes. Generally, in the manufacturing process, the composition of the mixture to be extruded is of capital significance and in order to get the desired physicochemical properties of the product. In that sense, they concluded that the addition of the natural parent clay as a binder in a ratio 80/20 and of water in a solid/liquid ratio of 0.53 seems profitable.

Dominguez *et al.*^64^ performed the deposition of Al–Fe pillared bentonite and gold supported Al/Fe pillared bentonite on metallic monoliths. The synthesized catalysts were characterized and their efficiency was investigated in the gaseous oxidation of CO as well as the oxidation of phenol in aqueous medium. They found that the deposition of pillared bentonite clay (with and without gold) was successful without changing the structural characteristics of the catalysts. The use of monoliths was found to enhance the catalytic activity in both oxidation reactions; however, more improvement was evident in the oxidation of phenol. The monolith assisted with the separation of the catalyst from the reaction medium in the liquid phase.

Han *et al.*^65^ synthesised a series of acidic montmorillonite/cordierite monolithic catalysts by a coating method using silica sol as the binder. The catalysts performances were investigated in the conversion of cumene hydroperoxide (CHP). They reported that acidic montmorillonite was homogeneously distributed on the surface of cordierite honeycomb monolith. The specific surface area of the cordierite honeycomb monolith considerably increased with an increase in the acidic montmorillonite. The catalysts were found to have a higher catalytic activity and selectivity towards conversion of CHP. It was also reported that this novel catalyst can be reused for several times without significant decrease in the activity.

Recently, Gatica *et al.*^66^ studied the use of pillared clays in the synthesis of washcoated clay honeycomb monoliths as support for manganese catalysts for the total oxidation of VOCs. Based on the physicochemical properties of the catalyst it was shown that the pillared clay played a positive role in optimizing the active phase deposited onto the surface of the honeycomb monolith surface by increasing its amount while keeping a relatively dispersed firmly bound active phase on the surface of the honeycomb monolith. The synthesized catalyst showed high efficiency and stability towards removal of the two model VOCs (acetone and propane) investigated. It was concluded that the data collected in their studies together with the intrinsic advantages of the honeycomb monolithic design, demonstrate the potential of the synthesised catalyst as a competitive material in the field of VOCs removal.

## 5. Catalytic application of PILCs catalysts in wastewater treatment

### 5.1 Overview

In the last decade of the 21^st^ century wastewater and water industries have come up with innovative trends focused on green and sustainable development.^67^ In general, sustainable technologies strengthen diverse products and services that deliver performance at lower costs, greatly reduce or remove environmental impact without forming secondary pollution and thereby improving the quality of life. Adsorbents and catalysts are significant for sustainable development in the green engineering wastewater industry. They aid in the treatment of wastewater contaminated by harmful substances in a resource protective way, with less energy usage and, in some cases, without any formation of harmful by-products and wastes. The catalysts, specifically if applied in a structured way, play a significant role in the so-called combined or integrated approach to environmental protection, such as integration of various wastewater treatment processes. These processes include separation, chemical reaction, momentum transfer and heat exchange. The result of such process integration is the decrease of investment costs, which is often combined with major energy recovery and space saving.

Recently, PILCs have been defined as porous materials of the 21^st^ century and have attracted much attention from both academic and industrial research point of view.^68–71^ Their high specific surface area, large micropore volume, upper acid site concentration, increased hydrophobicity and the increase in the density of sorption sites make them an excellent adsorbent and catalyst for removal of pollutants in wastewater.^4,71^ The removal of contaminants present in wastewater streams generated by various industrial processes has emerged as a significant concern during past decade.^72,73^ This is because of the decline in the amount and quality of available fresh water in the world due to the increase in water demands and long periods of drought. Many extremely toxic and hazardous compounds such as phenol and its derivatives are considered as persistent organic contaminants that occupy a prominent position on the WHO contaminants list owing to their high toxicity for microorganisms, high chemical oxygen demand and low biodegradability.^74^ Typical concentrations of phenol in industrial wastewaters are in the range of 200–1500 mg L^−1^ whilst the Environmental Protection Authority's limit for wastewater discharge are 0.5 mg L^−1^ for surface water and 1 mg L^−1^ for sewerage water.^74,75^ Therefore, effective removal of pollutants such as phenol in wastewater turns out to be a challenging task since environmental laws and regulations are becoming more stringent with time. Wastewater discharges such as phenols that are too dilute to incinerate and yet too toxic to be treated by biological process can suitably be dealt with by AOPs. AOPs have been defined as aqueous phase oxidation processes mainly based on the generation of highly active and non-selective hydroxyl radicals (HO˙) in its mechanism, that contribute to the breakdown of the organic pollutants in wastewater.^76^ The first step in the removal of organic contaminants by AOPs is adsorption, due to the fact that their mode of action occurs on the surface of the catalyst and both adsorption and AOPs processes would take place concurrently and the final removal would be due to the result of both removal by adsorption and AOPs. Low adsorption of these organic contaminants onto the surface of the PILCs, results in significantly low AOPs reaction rates. Consequently, increasing the adsorption capacity of organic contaminants onto PILCs may significantly increase the removal rate of organic contaminants.

In recent years, the modification of clay mineral to increase its adsorption capacity for the removal of contaminants from wastewater has been studied.^37,77,78^ Several research groups have focused on the improvement of the structural and textural properties of bentonite clays and their applications in wastewater treatment.^4,79,80^ Ding *et al.*^10^ prepared a novel Al/Cr-pillared montmorillonite clays materials and tested them for adsorption of benzene. The benzene adsorption capacity of the prepared Al/Cr-PILCs was found to be higher compared to the starting raw clay minerals. Furthermore, the results indicated that the material had excellent benzene adsorption performance, and complete desorption could be achieved at a temperature far below its calcination temperature, indicating that the material can be reused. Therefore, the prepared Al/Cr-PILCs were found to be good materials for adsorption. El Miz *et al.*^81^ synthesized Al-PILCs using Al_13_ [OH/Al = 2.4] from Moroccan clay and studied its aqueous thymol adsorption capacities using a batch equilibrium technique. The prepared materials were found to possess excellent adsorption capacity for aqueous thymol removal. The intraparticle diffusion plot in the study confirmed that the sorption process was controlled by particle diffusion. The amount of thymol released (desorbed) from the Al-PILCs was negligible in water. Mechanisms involved in the adsorption that describe the high thymol adsorption capacity and irreversibility, were the molecule fixed by the adsorbed clay on polar groups (Al–OH and Si–OH) (remaining) sites onto the basal plane and on the layer silicate edges. Owing to these properties, the Al-PILCs showed a high potential for use as an adsorbent for thymol in wastewater. Although adsorption seems to be an effective and simple method, the regeneration of the adsorbent increases the operational cost.^37,78–80^ In contrast, quite reasonable results are obtained in the removal of organic contaminants by AOPs as compared to adsorption due to the fact that hydroxyl radicals are not selective and have high oxidation capacity.^76^

Recently, Tomul *et al.*^37^ synthesized Ti-pillared bentonite, Cu, Ag and Fe modified Ti-pillared bentonite and Cu/Ti- and Fe/Ti pillared bentonite composites using different Ti sources *via* direct synthesis or by modification after synthesis. The synthesized materials were investigated for both adsorption and oxidation of aqueous bisphenol A. The bisphenol A adsorption capacity of the Fe/PTi-PILCs sample was observed to be higher compared to other samples. The adsorption of bisphenol A by the pillared bentonite tested was found to fit the Langmuir isotherm. On the other hand the PTi-PILCs, Cu/PTi-PILCs and Fe/PTi-PILCs, values close to complete bisphenol A conversion were obtained within 30 minutes of reaction time while the conversion rate was only 85% in the Ag/PTi-PILCs sample for the same reaction time. It was concluded that the synthesized pillared bentonite materials were effective for both adsorption and oxidation of bisphenol A from aqueous solution. Tomul^4^ synthesized the Fe/Cr-mixed bentonite pillared clay and investigated its activity for the oxidation of phenol. It was found that the temperature have major effect, compared to other studied parameters, increasing both the phenol oxidation and TOC removal while also retarding iron and chromium leaching from Fe/Cr-pillared bentonite catalyst. The iron and chromium leaching was not significant as were reported to be less than 0.71 and 1.13 mg L^−1^, respectively. These values were found to be less than the maximum allowed by the legislation for these metals.^74^ Moreover, during the oxidation of phenol, aromatic intermediates such as catechol, hydroquinone and benzoquinones were obtained and then it was observed that these intermediates are oxidized to organic acids such as oxalic acid, formic acid, maleic acid and fumaric acid.

Guo and Al-Dahhan^29^ evaluated extrudates of Al/Fe-PILCs in the packed-bed operations for wastewater treatment by a wet oxidation process. The oxidation of phenol in aqueous solution was investigated using a semi-batch reactor under mild conditions. The Al/Fe-PILCs attained complete oxidation of phenol and high removal of TOC. Intermediate products such as catechol, benzoquinone, hydroquinone, oxalic acid and acetic acid were identified. The catalyst was found to be reusable for several reaction cycles without any significant change in its catalytic activity with low amount of Fe leached from the catalyst into solution. The as-synthesized Al/Fe-PILCs was thus found to be a promising catalyst for industrial wastewater treatment. Nikolopoulos *et al.*^82^ investigated the removal of phenol using extrudates prepared with pillared bentonite clay at room temperature and atmospheric pressure. Extrudates with B-Al/Fe and B-Al/Ce/Fe were reported to attain total removal of phenol and TOC between 30 and 62%, in 9 hours reaction time. Thereafter, the catalyst was removed from the solution and the leachate was recovered and analysed, indicating Fe values in the range of 0.11–0.14 mg L^−1^. The differences in phenol and TOC removal time for the extruded materials compared to the powdered materials were attributed to a consequence of the agglomeration process and the inherent diffusional limitations. The extruded catalyst retained its catalytic activity during at least 10 consecutive tests (80 hours).

Sanabria *et al.*^21^ synthesized extrudates developed from Al/Fe and Al/Ce/Fe pillared bentonite shaped as pellets, Raschig rings and monoliths with Na- or Ca-bentonite as binder. The Al/Fe and Al/Ce/Fe-PILCs were investigated as catalyst in the removal of phenol. The synthesized extrudate materials were reported to maintain their chemical and catalytic properties of the synthesized materials in a powder form. The observed differences in the reaction time required to achieve similar phenol and TOC removal, were attributed to diffusional limitations inherently present in the extrudate materials.

### 5.2 Advanced oxidation processes

AOPs have gained recognition as alternative wastewater treatment processes against conventional methods, targeting the removal of non-biodegradable and toxic organic compounds.^83,84^ In general, the main objective of any AOPs design is to produce and use hydroxyl free radicals as strong oxidants to destroy chemical compounds which cannot be removed by conventional oxidants. AOPs technology has gained popularity as an imperative technology for oxidation and mineralization of refractory compounds in wastewater.^67^ Oxidizing power is one of the most important factors to be considered when selecting an oxidant for oxidation processes.^84^Table 1 shows the relative oxidation potentials of several chemical oxidizers. The HO˙ is the second strongest oxidant (second highest powerful oxidant after the fluorine) with a relative to oxidation power of 2.86 eV. These radicals are produced by means of oxidizing agent such as H_2_O_2_ and O_3_, ultrasound, ultraviolet irradiation, and homogeneous or heterogeneous catalysts. The HO˙ radicals are non-selective in nature and they can react without any other additives with a wide range of contaminants and attack organic compounds by either abstracting a hydrogen atom or adding hydrogen atom to the double bonds.^85,86^ The HO˙ radicals oxidize organic compounds to lower molecular weight intermediates or carbon dioxide and water in case of complete mineralization.^86^

**Table 1:** Oxidation potentials of some commonly oxidants

Substance	Potential (eV)
Fluorine (F_2_)	3.06
Hydroxyl radical (HO˙)	2.86
Oxygen (O)	2.42
Ozone molecule (O_3_)	2.07
Hydrogen peroxide (H_2_O_2_)	1.78
Oxygen molecule (O_2_)	1.23

AOPs include wet air oxidation (WAO), photocatalysis, catalytic wet peroxide oxidation (CWPO), catalytic wet air oxidation (CWAO) and Fenton processes.^30^ In recent decades, these methods have been applied successfully for the removal of recalcitrant pollutants based on the high oxidative power of the HO˙ radical.^4,86,87^ These methods are promising in converting toxic and biologically resistant compounds into less harmful intermediates compounds or carbon dioxide and water. However, most noticeable drawback of AOPs is their operating cost compared to other conventional physicochemical or biological treatments. This is due to the use of catalysts that are sensitive to poisoning and expensive. Therefore, AOPs cannot be easily implemented to industrial scale to achieve complete mineralization of non-biodegradable compounds due to this limitation. One of the most reasonable solutions to this problem is the use of abundant, inexpensive and environment friendly catalyst such as PILCs or coupling AOPs with less expensive conventional treatment methods. This process is justified commercially in the industrial application if the used catalyst can be less expensive, non-poisonous, reduce the severity of the operating conditions and completely mineralize the organic compounds. PILCs have been applied in AOPs as a catalyst or catalyst support due to their stability, structure, abundant availability, and cost-effectiveness. The use of PILCs in AOPs as a catalyst that helps in the removal of high toxic organic compounds has been frequently reported in the literature with promising selectivities suitable for various organic contaminants.^51,88–91^

#### 5.2.1 Photocatalysis

Heterogeneous photocatalysis is one of the AOPs technologies which is expected to develop to industrial scale in the near future. This is due to the fact that it does not need any chemicals addition, it is suitable for treating wastewater with low concentrations of organic pollutants, it is non-specific, and it can lead to the total mineralization of organic compounds.^92^ This technology can use solar energy, which can be of particular interest for countries with prolonged periods of sunlight.^93^ Heterogeneous photocatalytic process is one of the most important AOPs and it is based on the oxidation of organic compounds present in wastewater by means of a photochemical reaction occurring on a semiconductor catalytic solid surface activated by light with a specific wavelength. The use Ti-PILCs has attracted increasing interest because of their low toxicity, such kind of materials are being used as photocatalysts for efficient treatment of wastewaters containing toxic organic compounds.^92^ The photocatalytic oxidation of different organic compounds such as phenol, 4-chlorophenol, dimethyl phthalate ester and dyes using Ti-PILCs catalysts has been reported.^94–99^

However, most of the above mentioned studies reported photocatalytic activities of Ti-PILCs to be low compared to commercial available P25 TiO_2_. The possible reasons are related to lower amount of TiO_2_ in Ti-PILCs compared to pure TiO_2_, the difficulty in obtaining well crystallized nanocrystals, and the quantum-size effect, due to small TiO_2_ nanocrystals present (a blue shift in the absorption edge). Moreover, it appears that doping of the Ti-PILCs with silver decreases the catalytic activity in agreement with its lower adsorption capacity. Another most common problem associated with the photocatalysis process is the reduced efficiency of Ti-PILCs photocatalyst with continuous operation possibly due to the adsorption of contaminants at the solid surface and blocking of the UV activated sites, which makes them unavailable for the destruction of targeted organic compounds and the need to treat highly diluted wastewater. Therefore, the practical applicability of photocatalysis using Ti-PILCs in industrial scale is not eco-friendly. However, many researches at laboratory scale to date are investigating ways which are aimed at improving the process to escalate it to industrial scale. The use of natural sunlight is significant compared to the use of artificial UV lamps which are reported not feasible in the photocatalytic process. Therefore, the current need is to study how to use the PILCs catalysts structure to promote catalytic activity under sunlight and also to apply the hydrophobic micro environment of PILCs to absorb the non-polar pollutants.

So far, only few studies have reported the use of PILCs catalyst structure as heterogeneous catalyst to promote sunlight activity. Yang *et al.*^100^ and Zhang *et al.*^101^ prepared a composite TiO_2_-PILCs photocatalyst for the removal fuchsine and 4BS dye under sunlight. The photocatalytic activities in both studies were found to be comparable to that of commercial Degussa P25 TiO_2_ using sunlight irradiation. However, it was reported that the use of dyes adsorbing in the visible region is not appropriate valuation to study the photocatalytic properties of PILCs materials in the visible region, due to the dye self-sensitizer effect in the visible region.

#### 5.2.2 Photo-Fenton

Fenton peroxidation process has a long-established reliability to generate the powerful oxidant, hydroxyl radicals (HO˙) and is judged to be effective in the elimination of organic compounds in wastewater.^102^ In addition, it is relatively environmentally friendly due to the fact that it does not involve the use of harmful chemical reagents such as Fe^2+^ ions and hydrogen peroxide (H_2_O_2_) and the generated hydroxyl radicals (HO˙) are nontoxic.^103,104^ However, the conventional homogeneous Fenton processes require the removal of the ferric hydroxide sludge after wastewater treatment and it requires acidic conditions, typically below pH 3.0. Additionally, separation and reuse of these homogeneous catalysts are rather difficult at the end of the Fenton oxidation treatment process. These drawbacks limit the application of this process in the industrial scale, due to the high costs involved in terms of labour, reagents, acidification during processing and neutralization after treatment and time.^89^ To overcome these drawbacks, much attention has been paid to the development of immobilized Fenton catalysts, such as heterogeneous Fenton-like catalysts, which can promote a heterogeneous Fenton process.

Since the beginning of the 21^st^ century, efforts have been made to improve heterogeneous processes such as iron supported on materials like clays.^88,105–109^ PILCs have been commonly used in heterogeneous photo-Fenton applications.^110^ This is due to the low cost and abundance of clay minerals, together with the ease of the pillaring process, resulting in the successful immobilization of iron ions on the surface of a material with relatively larger specific surface area. Therefore, pillared clay catalysts have been employed in the photo-Fenton removal of organic contaminants such phenol and its derivatives,^107,108^ organic dyes,^105^ toluene,^111^ tyrosol^112^ and other persistent compounds.^113,114^ The catalyst physiochemical characteristics, as well as the operational parameters used in a specific reaction are reported to have a strong influence on the removal efficiency of organic contaminants in wastewater. As a result, it is essential to assess the effect of these factors on the effectiveness of the PILCs catalysts for removal of organic pollutants using heterogeneous Fenton process.

The first study which investigated the performance of pillared laponite clay-based Fe nanocomposites in the photo-Fenton (H_2_O_2_ + UV radiation) mineralization of azo-dye Acid Black was reported by Sum *et al.*^115^ They achieved a 100% mineralization of azo-dye Acid Black, whereas only trace amounts of leached Fe ions were detected. The study by Li *et al.*^89^ investigated the photo-Fenton removal of an organic azo-dye (Orange II) using Fe-pillared bentonite. They compared the reaction rates of the heterogeneous photo-Fenton process and homogeneous photo-Fenton process, in which it was found that the heterogeneous photo-Fenton process was much faster. It was further shown that the synthesized catalyst materials can also be easily recovered, regenerated, and re-used in the process. The photo-Fenton oxidation of Methylene Blue using Fe-PILCs catalysts with two different sized particle fractions (<250 μm and within the range of 250–450 μm, respectively) was studied by De León *et al.*^106^ Both PILCs catalysts performed very well for the removal of the dye from aqueous solution, but the differences in catalyst performance were linked to textural properties. Recently, Bel Hadjltaief *et al.*^109^ synthesized an iron-pillared Tunisian clay (Fe-PILCs) and used as the catalyst in the heterogeneous photo-Fenton oxidation of Red Congo and Malachite Green in aqueous solution. They found that both dyes were successfully removed from the aqueous solutions using a heterogeneous photo-Fenton process in the presence of Fe-PILCs as the catalyst. Furthermore, stability and leaching tests showed that the catalyst was stable over the evaluated reaction time and cycles.

Heterogeneous Fenton oxidation using PILCs has been demonstrated in full laboratory scale applications as a feasible technology for the treatment of a wide diversity of organic pollutants in aqueous solutions. However, many studies were carried out using H_2_O_2_ and UV light, which is not economically feasible at a commercial scale. Therefore the possibility of using sunlight is necessary for commercial scalability. The economy of this technology is very dependent on the H_2_O_2_ consumption; and therefore safe handling of H_2_O_2_ and minimal usage of H_2_O_2_ when dealing with high organic loads are important considerations.

#### 5.2.3 Catalytic wet peroxide oxidation (CWPO)

CWPO processes using H_2_O_2_ (hydrogen peroxide) as the oxidant instead of oxygen have emerged as a feasible alternative for the wastewater treatments of medium-high refractory pollutants and TOC concentrations.^116^ This process operates at milder reaction conditions (temperature lower than 100 °C and atmospheric pressure) and does not need expensive reactors, offering significantly lower operative and fixed capital costs. The absence of a gas/liquid boundary in the CWPO process removes mass-transfer limitations and the H_2_O_2_ acts as a free-radical initiator, providing radicals (HO˙) that promote the removal of organics pollutants in wastewater. This results in reduction of reaction times and allows conversion under milder reaction conditions. However, it should be noted that H_2_O_2_ is a more expensive reactant compared to oxygen (air). One of the major drawbacks of this process is the instability of the solid catalyst due to the chemical leaching of active metal such as Fe and Cu into the liquid phase of reaction. In order to increase the breakdown of H_2_O_2_ to hydroxyl radicals the use of an inexpensive, stable, ecofriendly and superior activity catalysts is highly desirable.

Among different solid catalytic materials that have been used in CWPO process, PILCs are receiving increasing attention.^8,117^ Therefore, these materials can be used as a new class of micro- and mesoporous materials for catalytic applications in both acid–base and oxidative reactions.^8,118,119^ PILCs with mixed oxides of aluminium/iron or aluminium/copper have been seen as a feasible option for the removal of organic pollutants such as dyes and phenolic compounds from wastewaters.^8,119^ PILCs catalysts have been reported by Galeano *et al.*^3^ to achieve high rates of pollutants removal with a very high stability and a minimal leaching of the interlayered metal species.^7,118,120^ Garrido-Ramírez *et al.*^120^ and Zhou *et al.*^7^ reported that PILCs can also perform over a wide range of pH and temperature and can be readily separated from the aqueous solution and retain their catalytic activity in repeated cycles. They reported relatively short operation times owing to the high catalytic activity of the PILCs.

Barrault *et al.*^121^ were amongst the first to study the removal of phenol in wastewater under mild reaction conditions (70 °C and atmospheric pressure) using mixed Al/Fe-PILCs in CWPO. Their study showed that approximately 80% of the phenol present was converted into CO_2_ and H_2_O within 2 hours reaction time. The stability of Al/Fe-PILCs catalyst was reported to be superior, even after three cycles of the reaction and only less than 1 mg L^−1^ of iron leached to the solution. Barrault *et al.*^121,122^ were also amongst the first researchers to report the good performances of mixed Al/Cu-PILCs synthesized from a crude bentonite clay mineral in phenol oxidation with H_2_O_2_. They reported that the introduction of copper in pillaring position resulted in more effective catalysts, although the amount of copper which can be introduced was limited to less than 0.5 wt%. This catalytic material was reported to be stable against leaching and the catalyst was reusable in consecutive batch reactor tests. Caudo *et al.*^123^ compared Cu-PILCs and Fe-PILCs in the CWPO of both model phenolic compounds (*p*-coumaric and *p*-hydroxybenzoic acids) and real olive oil milling wastewater. The synthesized solid materials exhibited similar performances in all these reactions, although Cu-PILCs showed a lower formation of oxalic acid (main reaction intermediate) compared to Fe-PILCs. Both catalysts showed a high resistance to leaching of active metal and a good catalytic performance.

The first study to investigate the removal of the refractory contaminants in the coffee wet processing wastewater (CWPW) produced in Colombia using CWPO was reported by Peralta *et al.*^124^ In their study the oxidation of the phenolic compounds in CWPW was examined using Al/Fe-PILCs extrudates which were shaped into Raschig rings. The investigation was conducted at mild operating conditions (temperature of 25 °C and atmospheric pressure), the Al/Fe-PILCs extrudates were found to be effective catalysts for the removal of phenolic compounds and TOC reduction in CWPW. The catalyst extrudates were reported to have achieved a 62.4 and 67.5% phenol removal and selectivity toward CO_2_ after 96 hours of the reaction time. Moreover, the Al/Fe-PILCs extrudates showed superior catalytic activity during eight successive oxidation experiments without significant Fe leaching. Therefore, the use of Al/Fe-PILCs extrudates in the removal of phenolic compounds demonstrated the potential for treatment of real wastewaters contaminated by phenolic compounds. This study showed a positive contribution to the eco-friendly and sustainable development in the use of CWPO for real wastewater treatment. This is mainly due to the fact that the Al/Fe extrudates materials can keep the properties of the pillared clay (high specific surface area and reactivity), show good mechanical stability and do not disintegrate upon contact with real wastewater. In addition, the expensive separation step (filtration or centrifugation) is not compulsory as the extrudates can be easily separated from wastewater after reaction, unlike powder solids catalysts materials. Therefore, these features make PILCs catalyst extrudates an attractive alternative for treating wastewater using CWPO.

In summary, several studies showed interesting and stable performances of PILCs-based catalysts for CWPO process. They can be used for practical applications, although studies on their practical industrial application in CWPO are limited, as most of the studies in the literature only focus on the use of model compounds. However, up to date there are few commercial CWPO processes using solid catalysts materials. These include the OXY-PURE of Delta Umwelttechnik which is developed to remove cyanide, phenols and other organic species from heavily organic loaded and turbid wastewaters and the USP Technologies (formerly known as US Peroxide processes) for treatment of municipal wastewater, drinking water treatment and industrial wastewater.^124^

#### 5.2.4 Catalytic wet air oxidation (CWAO)

Among the methods employed to oxidize recalcitrant organic contaminants, the CWAO is of significant interest due to its high oxidizing ability and complete mineralization.^125^ The CWAO is a liquid-phase oxidation process using a solid catalyst in which organic and inorganic compounds, present in the solution, is oxidized by oxygen or air basically to CO_2_ and H_2_O, without any toxic gaseous emissions such as NOx, SO_2_, HCI, dioxins, furans, fly ash, *etc.* CWAO is considered an attractive and useful method for treatment of organic contaminants effluents with concentrations which are too low for incineration and too high for biological treatment, *e.g.* in the case of harmful organic effluent such as phenol.^71^ The incineration process is reported to be uneconomically feasible due to additional fuel cost or when heat recovery would result in only marginal saving.^126^ The reaction is typically carried out at elevated temperatures (200–325 °C) and pressures (5–20 MPa) in order to optimize the oxygen concentration in the dissolved phase and consequently improve the CWAO process efficiency.^126^ Although, CWAO has shown excellent potential to treat industrial wastewater, its application is limited due to the high capital investment and the high operating costs involved.^127^ This is due to the severity of operating conditions and the lack of stable and active catalysts, which prevent a wider industrial commercialization. Therefore, many research efforts have been devoted to decrease the severity of the operating conditions through the development of appropriate solid catalysts.^127–129^ If a stable solid catalysts can be developed that works effectively below 125 °C and 2.5 MPa, CWAO would be a potential wastewater treatment process for more general water treatment.^130^ The use of PILCs catalysts in the CWAO has been proposed as this can effectively decrease the severity of the operating conditions, shorten reaction time and enhance the oxidation efficiency of organic compounds.^29^

Guo and Al-Dahhan *et al.*^29^ studied the use of PILCs catalyst in the CWAO of aqueous phenol solution. The CWAO kinetics of an aqueous solution of phenol was investigated using Al/Fe-PILCs extrudates (cylindrical, approximately 2 × 8 mm) in the temperature range of 90–150 °C and air pressure range of 0.8–2.5 MPa. The reaction temperature, air pressure, initial solution pH, initial phenol concentration, and catalyst loading were the operating parameters studied. The employed conditions in their studies were much milder compared to typical industrial operating conditions. The comparison between results obtained without hydrogen peroxide and using hydrogen peroxide oxidation were done in this study. This is one of the few studies in open literature wherein a good comparison between the performances in catalytic oxidation with wet air and H_2_O_2_ (CWAO and CWPO, respectively) of the same catalyst was investigated. In the presence of H_2_O_2_ the Al/Fe-PILCs was reported to have attained a total removal of phenol and significant mineralization (80–100%) of TOC conversion without significant Fe leaching and catalyst deactivation. However, the cost and noticeable corrosion issues associated with H_2_O_2_ restricted their efforts on H_2_O_2_ oxidation and focused on the oxidation by air only. The total removal of phenol by CWAO was achieved at optimum pH of 4 and low pressure of 1.5 MPa. The optimum temperature was found to be between 130 and 150 °C, above which the operating pressure was reported to exceed the vapour pressure of the liquid phase which was uneconomical. The deactivation of the Al/Fe-PILCs extrudates catalyst due to fouling and Fe leaching was reported to be insignificant, although the solution was acidic and at high temperature. Therefore, the PILCs catalyst was found to be stable and maintained its activity for long-term experimental reaction.

The development of commercial CWAO processes based on homogenous catalysts (*e.g.*, Ciba-Geigy, LOPROX, WPO, ORCAN and ATHOS processes) started as early as the mid-fifties in the United States.^131,132^ Homogeneous transition metal catalysts based on Cu^2+^ or Fe^2+^ ions are currently used in numerous commercial CWAO plants, which are operating successfully to treat different industrial discharges and residual sludge. Table 2 summarises some commercial CWAO processes. Ciba-Geigy was the first to commercialize CWAO process that applies a copper salt as a catalyst. Currently, there are three plants operating in Germany and Switzerland, these plants are dedicated to the treatment of chemical and pharmaceutical wastes. These plants operate at 300 °C and pressure above 15 MPa and have accomplished high oxidation efficiencies (95–99%) of chemical and pharmaceutical wastewaters. However, there is a need to remove the homogeneous transition metal catalysts from wastewater stream after treatment in a subsequent step and recycle to the reactor inlet or discard it. On the other hand the heterogeneous catalysts can be separated from treated wastewater easily and reused, while maintaining their catalytic activity for sufficiently long periods.

**Table 2:** Commercial CWAO processes for the treatment of industrial wastewater^131–133^

Process	Catalyst/oxidant	Temperature, °C	Pressure, MPa	Application
LoProxBaye	Fe^2+^-acid/O_2_	<300	0.5–2	Chemical/pharmaceutical waste
Ciba-Geigy	Cu^2+^/air	∼300	∼15	Chemical/pharmaceutical waste
Athos	Cu^2+^/O_2_	235–250	4.4–5.5	Residual sludge
WPO	Fe–Cu–Mn/H_2_O_2_	90–130	0.1–0.5	Aquifer decontamination
Orcan	Fe^2+^/air + H_2_O_2_	120	0.3	Refractory waste pre-treatment
The Nippon Shkubai (NS-LC)	TiO_2_ supported multi-metal catalysts (oxides of lanthanide series and transition metals)	160–270	0.9–8	Can treat acetic acid, phenol, formaldehyde and ammonia
Osaka gas	ZrO_2_ or TiO_2_ with noble or base metals (Fe–Co–Ni–Ru–Pd–Pt–Cu–Au–W)	250	6	High COD and ammonia containing wastewaters
Kurita	Supported Pt	170	—	Ammonia
CALIPHOX	CuO–ZnO–Al_2_O_3_	180	4	Industrial wastewaters

Therefore, three commercial heterogeneous CWAO processes (*e.g.* NS-LC, Osaka Gas and Kurita) have been developed in Japan since mid-eighties.^133^ These heterogeneous CWAO processes are based on heterogeneous catalysts containing precious metals deposited on titania or titania–zirconia supports and are able to oxidize different refractory compounds, therefore, allowing the treated wastewater to be discharged directly to water bodies or reused as process water. The NS-LC process uses a vertical monolith reactor with a Pt/Pd/TiO_2_–ZrO_2_ honeycomb heterogeneous catalyst. The operating conditions are in the range of 160–270 °C and 0.9–8 MPa with space velocity of 2 L h^−1^ and can treat different organic effluents such as acetic acid, phenol, formaldehyde and ammonia, up to more than 99% conversion. The Osaka gas process applies a combination of precious and base metals on titania or zirconia–titania supports (Fe–Co–Ni–Ru–Pd–Pt–Cu–Au–W) in a form of honeycomb or sphere as heterogeneous catalyst. Typical operating conditions are 250 °C and 6 MPa, with 200 L h^−1^ feed of wastewater and this process targets wastewater effluent containing high COD and ammonia. The Kurita process uses nitrite as oxidant instead of oxygen in the presence of a supported Pt catalyst. This process is more effective at lower temperatures, around 170 °C and is used to treat wastewater effluent containing ammonia. The CALIPHOX process is the most recently developed process to treat industrial wastewaters using metal oxide catalyst extrudates in a trickle-bed reactor. This process was developed by the National Institute of Chemistry of Slovenia and an engineering firm and operates at relatively mild conditions (180 °C and 4 MPa).

In conclusion, as it can be observed from Table 2, there have been more CWAO commercialised processes based on homogenous catalysis than heterogeneous catalysis. Moreover, the industrial applications of CWAO operate at temperatures and pressures that are not considerably low compared to those used in non-catalytic wet oxidation. This observation indicates the urgent need for the development of more active, eco-friendly, cheap and stable heterogeneous catalysts, which can reduce the severity of the reaction conditions. However, it is also interesting to note that majority of these heterogeneous processes have used transition metal catalysts either as sole active component or in combination with other active components instead of expensive noble metals.

## 6. Conclusions and future prospects

The application of pillared clays as heterogeneous catalysts offers a variety of potential benefits in AOPs wastewater treatment technologies, from both the sustainable green chemistry and engineering perspective. However, it is essential to further advance background knowledge to completely understand limitations and advantages towards commercialization. The current reviewed results are promising to fill some specific gaps, which prevent the process to be escalated to industrial scale. Up to date however, no industrial examples of the use of pillared clays as catalysts in AOPs exist. The next step involves a more rational design and engineering of catalysts materials and reactors. Along this review, important aspects of AOPs technologies have been reviewed and studies on treatment of wastewaters on the industrial scale/commercialized processes demonstrate the potential of these technologies. The possible benefits such as mineralization of highly toxic refractory compounds, mild reaction conditions and shorter residence times and the formation of harmless products, from AOPs specifically CWAO process, will be a key driver for more research studies in the field. The main current problem in the development of successful industrial-scale heterogeneous CWAO process for the treatment of industrial wastewater seems to be the design of appropriate catalyst that is highly active, stable, economical, easy to recover, reusable and environmentally friendly. Improvements to overcome high operational costs of CWAO process offer attractive alternatives, such as PILCs materials as inexpensive robust catalysts for CWAO of refractory organic contaminants. Therefore, further research in the area of catalyst development, with the focus on the low cost, catalyst stability, reusability and environmental friendliness such as PILCs, will lead to the discovery of solids heterogeneous catalysts that are effective for many industrial-scale CWAO processes. The use of novel green synthesis methods such as microwave radiation and ultrasonic treatment has proven to be effective methods with more improved physicochemical properties and high catalytic activity of PILCs compared to conventional methods. The new developments in the synthesis of PILCs substantially mitigate deactivation of catalysts due to leaching, long period of synthesis and high water consumption, which are the most important drawback in industrial scale application of wastewater treatment.

## Conflicts of interest

On behalf of all authors, there are no conflicts to declare.

## Supplementary Material
